# De novo assembly, annotation and gene expression profiles of gonads of Cytorace-3, a hybrid lineage of *Drosophila nasuta nasuta* and *D. n. albomicans*

**DOI:** 10.5808/gi.20051

**Published:** 2021-03-09

**Authors:** Koushik Ponnanna, Stafny M. DSouza, Nallur B. Ramachandra

**Affiliations:** Department of Studies in Genetics and Genomics, University of Mysore, Mysuru 570006, India

**Keywords:** divergence, *Drosophila*, *Drosophila nasuta albomicans*, *Drosophila nasuta nasuta*, homoploid hybrid, transcriptomics

## Abstract

Cytorace-3 is a laboratory evolved hybrid lineage of *Drosophila nasuta nasuta* males and *Drosophila nasuta albomicans* females currently passing ~850 generations. To assess interracial hybridization effects on gene expression in Cytorace-3 we profiled the transcriptomes of mature ovaries and testes by employing Illumina sequencing technology and *de novo* transcriptome assembling strategies. We found 26% of the ovarian, and 14% of testis genes to be differentially expressed in Cytorace-3 relative to the expressed genes in the parental gonadal transcriptomes. About 5% of genes exhibited additive gene expression pattern in the ovary and 3% in the testis, while the remaining genes were misexpressed in Cytorace-3. Nearly 772 of these misexpressed genes in the ovary and 413 in the testis were either over- or under-dominant. Genes following *D. n. nasuta* dominance was twice (270 genes) than *D. n. albomicans* dominance (133 genes) in the ovary. In contrast, only 105 genes showed *D. n. nasuta* dominance and 207 showed *D. n. albomicans* dominance in testis transcriptome. Of the six expression inheritance patterns, conserved inheritance pattern was predominant for both ovary (73%) and testis (85%) in Cytorace-3. This study is the first to provide an overview of the expression divergence and inheritance patterns of the transcriptomes in an independently evolving distinct hybrid lineage of *Drosophila*. This recorded expression divergence in Cytorace-3 surpasses that between parental lineages illustrating the strong impact of hybridization driving rapid gene expression changes.

## Introduction

The *nasuta* subgroup of *immigrans* species of *Drosophila* representing over nominal 12 species or subspecies exhibits varying degrees of the post and pre-zygotic isolations, making it a potent cluster to study speciation genetics. Comparative investigations among the members of *nasuta* radiation have provided insights into cellular complexity at genomic and transcriptomic levels. The evolutionary history of the rapidly radiating *nasuta* subgroup is well characterized, which has allowed for taxonomic inferences [1]. Genome-wide studies have revealed early stages of sex chromosome evolution and the origin of B chromosome in *D. albomicans* [2]. Transcriptome studies in *D. nasuta* and *D. albomicans* have formed the basis for deducing the role of alternative splicing towards lineage-specific evolution in *Drosophila* [3]. *Drosophila nasuta nasuta* (*D. n. nasuta*) and *Drosophila nasuta albomicans* (*D. n. albomicans*) belong to the orbital sheen complex, one of the three sheen complexes characterized in the *nasuta* subgroup of *Drosophila* [4]. Despite the documented initial post-zygotic incompatibilities in the F1 hybrids, the combinatorial crossing of various strains of these karyotypically diverged species and their successive maintenance has resulted in an assemblage of stabilized hybrids termed cytoraces [5,6]. In nature, many instances of hybridization in *Drosophila* are reported [7]. However, the number of laboratory-induced interspecific hybrids between closely related *Drosophila* species is relatively high [8].

**Fig. 1.**
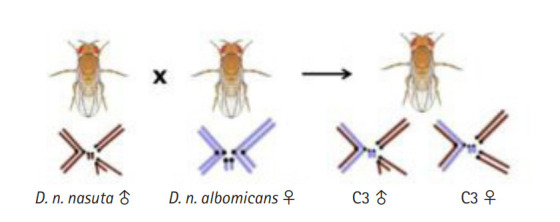
Illustration of the cross between *Drosophila nasuta nasuta* males and *D. nasuta albomicans* females leading to the formation of laboratory evolved interracial hybrid, C3 with a stable chromosome complement of 2n = 8 resembling *D. n. nasuta* with 10 *D. n. nasuta* and six *D. n. albomicans* chromosomes. C3, Cytorace-3.

Cytoraces with their parental species constitute the *nasuta-albomicans* complex (NAC) of *Drosophila*, which is the longest ongoing evolutionary experimentation in a laboratory setting in the genus *Drosophila* and the independently evolving populations of cytoraces are currently passing ~850 generations [9]. Cytorace-3 (C3), a member of NAC stemmed from the interracial hybridization event between *D. n. nasuta* males (Coorg strain), and *D. n. albomicans* (Thailand strain) females carry a stable chromosome complement of 2n = 8 resembling *D. n. nasuta* with ten *D. n. nasuta* chromosomes and six *D. n. albomicans* chromosomes (Fig. 1). The rapid divergence in C3 is extensively recorded through comparative studies for male and female reproductive fitness, wing size, genitalia size, body weight, body size, longevity [10], mating latency, and copulation duration in heterogamic crosses [11]. This empirical evidence makes C3 an attractive model to study the molecular basis of early events in racial divergence.

Advances in RNA sequencing technologies and bioinformatics approaches for data analysis have enabled the precise measurement of gene expression levels. De novo transcriptome assembling is a widely used method for conducting spatio-temporal and condition specific gene expression profiling in non-model organisms in lieu of reference genomes. This dissemination of technology has allowed researchers to investigate evolutionary questions once limited to model organisms [12]. Recently, transcriptomics' increased applicability has permitted quantifying hybridization-induced effects at a transcription level in interspecific hybrids and pure species [13-16]. Interspecific hybrids display a wide range of genetic changes in the hybrid genomes, ranging from introgression, chromosomal rearrangements, and differential gene expression to epigenetic modifications [13-15,17-19]. Such genetic changes invariably provide an insight into fixed accumulated species-specific changes limiting transgenerational assessments in hybrids due to failed reproduction, restricting the formation of true-bred hybrid populations. Therefore, the extent of genetic divergence between species can influence the outcome of hybridization and is well argued in the context of *Drosophila* [20].

In this study, we examined the expression profiles of mature ovaries of virgin females and testes from naive males of C3 employing Illumina sequencing technology and *de novo* transcriptome assembling strategies. The study aims to comprehensively quantify gene expression divergence in C3 and deduce the inheritance of gene expression levels through comparative analysis with their parental transcriptomes. To our knowledge, this is the first report focused on a systematic exploration of expression profiles in the gonads of an independently evolving established homoploid hybrid lineage of *Drosophila*. The present findings amalgamate the existing works in understanding the onset of hybridization driven speciation and provide a valuable resource for subsequent studies.

## Methods

### RNA sequencing and *de novo* transcriptome assembly

**Fig. 2.**
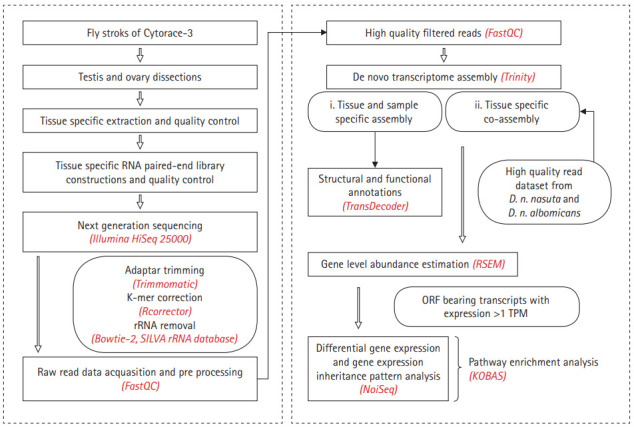
Schematic representation of the workflow. Flowchart of the pipeline for the C3 gonadal transcriptome sequencing, *de novo* assembly, annotation and differential gene expression analysis. C3, Cytorace-3; RSEM, RNAseq by Expectation-Maximization; TPM, transcript per million.

Fly stocks of C3 were maintained under laboratory conditions of 22±1°C, 12:12 LD cycle in standard wheat cream agar medium. Ovaries were dissected from 3-5 days old virgin females and testis from 3‒5 days old naive males. For each tissue type, 60 flies were dissected in ice-cold phosphate-buffered saline. To preclude mating induced transcriptional responses which are sex specific, the study focused on profiling only the gonadal transcriptomes in the virgin flies. RNA extraction was performed using the ZR-DuetTM RNA MiniPrepPlus kit (Zymo Research, Irvine, CA, USA), followed with cDNA libraries preparation using NEBNext Ultra RNA library prep kit for Illumina (New England Biolabs, Ipswich, MA, USA) and validated using Agilent DNA HS (Agilent Technologies, Santa Clara, CA, USA) and Qubit DNA BR assay kits (Life Technologies, Carlsbad, CA, USA). The sequencing of QC passed libraries was carried on the Illumina HiSeq 2500 (Illumina, San Diego, CA, USA) platform for 2 × 101 bp read length. Reads of parental lineages that belong to the same bio-project were downloaded from NCBI accessions SRR8398945 and SRR10875322 (*D. n. albomicans* ovary and testis), SRR8398946 and SRR10875323 (*D. n. nasuta* ovary and testis). A multistep raw data processing approach was undertaken to produce high quality read datasets (Fig. 2). The datasets were checked for sequence quality using FASTQC v0.11.5 (https://www.bioinformatics.babraham.ac.uk/projects/fastqc/). Subsequently, QC passed datasets were then corrected for low K-mer values using Rcorrector [21] followed by rRNA contamination removal using the SILVA rRNA database [22]. Following this, high quality read datasets from each library were *de novo* assembled to construct a tissue-specific transcriptome assembly for C3 and a co-assembled reference assembly of each tissue by pooling high quality reads from C3, *D. n. nasuta* and *D. n. albomicans* using Trinity v2.6.6 with default parameters [23]. The co-assembling strategy has been implemented in studies involving closely related species which have not diverged extensively. Considering divergence history of the parental lineages and C3 this strategy is deemed suitable in the present study and enhances the ability of the assembler to construct lowly expressed transcripts. Redundancy imparted in assembling stages was reduced by clustering transcripts with >95% similarity using CD-HIT-EST v4.6 [24].

### Structural and functional annotations of assembled transcriptomes

TransDecoder v5.5.0 [23] was run on clustered assemblies to extract open reading frames (ORFs) from transcripts and predict potential coding sequences using default parameters. Assignment of functional definitions to assembled transcripts was performed by querying against UniprotKB/Swiss-Prot database (release June 2019), EggNOG [25], Pfam v31.0 [26], TMHMM v2.0 [27], and SignalP v4.1 [28] databases and results compiled using Trinotate v3.1.1-pl526_5 [29] pipeline.

### Differential gene expression analysis of Illumina sequence data

Transcript abundance was estimated by aligning high quality reads used for assembly construction against the final co-assembled reference assembly using Bowtie2 v2.3.4.3 [30]. Following this, raw gene-level counts were estimated using RNAseq by Expectation-Maximization (RSEM) package [31]. A Gene with a potential ORF and with a minimum one transcript per million (TPM) value was considered as expressed and was used in downstream expression analysis. Pairwise comparisons were performed for differential gene expression analysis between C3 and *D. n. nasuta* and *D. n. albomicans* for ovaries and testis datasets using NOISeq-sim function of NOISeq v2.10.0 [32] with trimmed mean of M-values normalization. The values of log2 fold-change of 1 (positive or negative for either up or down-regulation respectively) with a probability of >0.9 was defined as significantly differentially expressed gene (DEG). Genes failing to fulfil this criterion were grouped as non-DEGs. From pairwise gene expression comparisons, six different expression inheritance patterns were derived. A gene was defined conserved if it was not differentially expressed in C3 and the parents. Misexpressed gene was defined as either over-dominant (gene for which expression was significantly higher in the C3 than both parents) or under-dominant (gene for which expression was significantly lower in the C3 than both parents) and additive (a gene differentially expressed in parents and showed intermediate expression in C3). Genes were classified as *nasuta* or *albomicans* dominant if a DEG in C3 exhibited similar patterns of expression with a corresponding parental lineage. Gene Ontology terms were retrieved using BLAST [33] best hits queried using UniprotKB/Swiss-Prot for these DEGs and plotted using WEGO v2.0 online tool [34], and Kyoto Encyclopedia of Genes and Genomes (KEGG) pathway enrichment analysis was performed using KEGG Orthology-Based Annotation System v3.0 (KOBAS) [35].

### Real-time quantitative polymerase chain reaction validation of DEGs

The reliability of the differential expression results quantified by whole transcriptome sequencing (WTS) was validated through real-time quantitative polymerase chain reaction (RT-qPCR). Twelve DEGs (six each from ovary and testis) which were commonly differentially expressed in C3 ovaries and testes relative to the parental transcriptomes were selected for validation. Primers were designed using Primer3 v0.4.0 software [36] and were synthesized at Xcelris Labs Ltd. (Ahmedabad, India). Gene list with primer sequence information along with respective Tm values is provided in Supplementary Table 1. Ovaries were dissected from 3‒5 days old virgin females and testis from 3‒5 days old naive males. Total RNA was extracted separately from ovary and testis tissues (30 pairs each) of the three samples using the GeneJET RNA purification kit (Thermo Fisher Scientific, Waltham, MA, USA) and quantified using Nanodrop 1000 spectrophotometer (Thermo Fisher Scientific). cDNA synthesis was carried by RevertAid first-strand synthesis kit (Thermo Fisher Scientific) in a 20 µL reaction volume according to the manufacturer's protocol. As a confirmatory step to check for the presence of expressed genes, an additional PCR (40 cycles of 95°C for 10 s, 60°C for 20 s, and 72°C) was carried using the cDNA with the selected primer pairs. Quantification was carried in biological triplicates and technical duplicates in StepOnePlus Real-Time PCR System (Applied BioSystems, Foster City, CA, USA) using SYBR Green chemistry (Maxima SYBR Green/ROX qPCR Master Mix 2×) (Thermo Fisher Scientific). The specificity of primers in amplifying target genes was reconfirmed by melt curve analysis. *Gapdh2* was used as an endogenous control following which relative gene expression quantification was calculated by the 2^-ΔΔct^ method.

### Accession numbers

The accession numbers for the C3 ovary and C3 testis sequences reported in this paper are deposited in Sequence Read Archive: SRR8398943, SRR10875320 under BioProject ID PRJNA512942 and PRJNA600771.

## Results

### Transcriptome sequencing and assembly construction

**Fig. 3.**
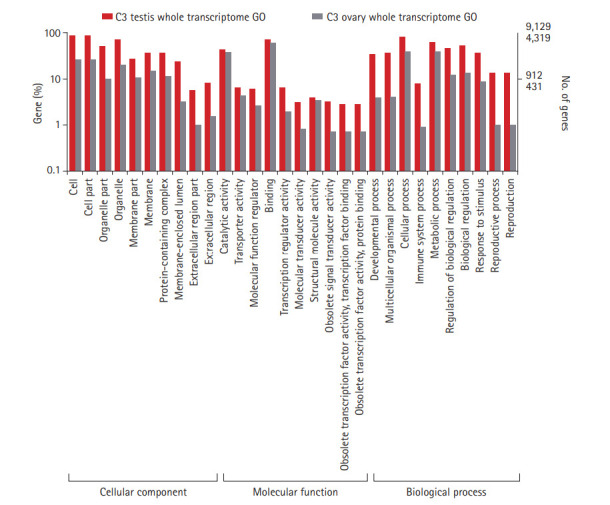
Gene ontology classification of annotated genes in C3 ovary and testis transcriptomes. Representation of top 10 GO terms at level 2 classification for the three functional categories. GO categories are shown in the X axis and percentage of genes in the Y axis (Log scale). C3, Cytorace-3; GO, gene ontology.

Tissue-specific sequencing of the C3 using Illumina Hiseq2500 paired-end yielded a total of 16,419,268 and 10,466,674 reads corresponding to ovarian and testes datasets. Sequence reads with PHRED score <20 were discarded. Subsequent removal of rRNA contamination, adaptor sequences, and K-mer correction generated 15,946,326 and 10,100,250 high-quality filtered reads for ovary and testis, further used for transcriptome assembly construction. Tissue-specific assemblies were constructed using C3 reads adopting the *de novo* approach with Trinity software. Tissue-specific assemblies were used to assess the coding potential of transcripts and for assigning functional definitions. We assembled 16,642 transcripts from ovarian, and 20,823 transcripts from the testis reads of C3. Redundancy removal through clustering resulted in the retention of ~92% and ~94% transcripts from C3 ovary and testis assemblies, respectively. Alignment rate of 96.92% of ovarian reads and 95.93% of testis reads deduced from back-mapping the reads to the constructed assemblies are indicative of good quality assemblies suitable for transcriptome characterization. Assembly statistics are provided in Table 1, and the functional annotation of the assembled transcripts against various databases is summarized in Table 2. Comparative enrichment analysis of gene ontology (GO) terms for the testis and ovary transcriptome of C3 was categorized into “biological process,” “molecular functions,” and “cellular component” (Fig. 3).

A combined reference assembly of 15,273 transcripts (ovarian co-assembly) was generated by pooling the high-quality filtered reads from ovaries of parental lineages and C3. Similarly, a testis-specific co-assembly (testis co-assembly) was also generated with 19,465 transcripts. Transcripts that carried a potential ORF and had a minimum expression of 1 TPM were labeled as "expressed" and considered for further analysis.

### C3 transcriptomes exhibit a pronounced expression divergence relative to the parental lineages over three decades of their formation

**Fig. 4.**
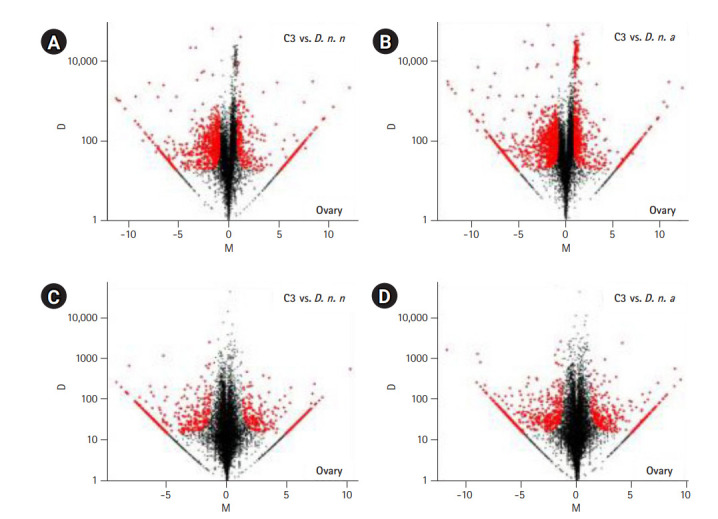
Pairwise differential gene expression analysis in C3 transcriptomes. Log2-fold change (M) is plotted on the X axis and the absolute value of the difference in expression (D) on the Y axis: C3 and *Drosophila nasuta nasuta* ovaries (A), C3 and *Drosophila nasuta albomicans* ovaries (B), C3 and *D. n. nasuta* testes (C), C3 and *D. n. albomicans* testes (D). Black points represent all statistically insignificant non-differentially expressed genes and red points represent significant differentially expressed genes. C3, Cytorace-3.

The expressed transcripts were further reduced to genes by retaining the longest transcript with a BLASTP hit to a known protein in the UniprotKB database. In total, abundance of 7,114 genes from the ovarian co-assembly and 8,673 genes from testis co-assembly was estimated. Pairwise differential gene expression was calculated for C3 testis and ovary against the parental samples. Fifteen percent of the genes were differentially expressed in C3 ovary in comparison to *D. n. nasuta* ovary (350 upregulated genes, 726 downregulated genes) while 21% were differentially expressed in comparison to *D. n. albomicans* ovary (581 upregulated genes, 941 downregulated genes). In testis of C3, 8% of the genes were differentially expressed in comparison to *D. n. nasuta* testis (293 upregulated genes, 389 downregulated genes) while 11% were differentially expressed in comparison to *D. n. albomicans* testis (383 upregulated genes, 568 downregulated genes). Overall, 26% of the C3 ovarian and 14% of C3 testis genes were differentially expressed (Fig. 4A-4D).

Among the pairwise comparisons, genes differentially expressed in C3 relative to both its parents were listed, and their KEGG enrichment analysis was performed. Two hundred twenty-five upregulated genes in C3 ovary constituted 48 KEGG pathways, of which, ten were significantly enriched. Five hundred forty-six downregulated genes in C3 ovary constituted 51 KEGG pathways, of which, seven were significantly enriched. 169 upregulated genes in C3 testis constituted 59 KEGG pathways, of which, ten were significantly enriched. Two hundred forty-four downregulated genes in C3 testis constituted 143 KEGG pathways, of which, 56 were significantly enriched. A detailed list of these enriched KEGG pathways is provided in Supplementary Table S2-S5. Enrichment analysis of GO terms on these common DEGs was performed, and the top ten GO terms from the three categories, “biological process,” “molecular functions,” and “cellular component,” are shown in Supplementary Figs. 1-4.

### Gene expression inheritance analysis indicates the predominance of conserved levels of expression in the hybrid lineage

**Fig. 5.**
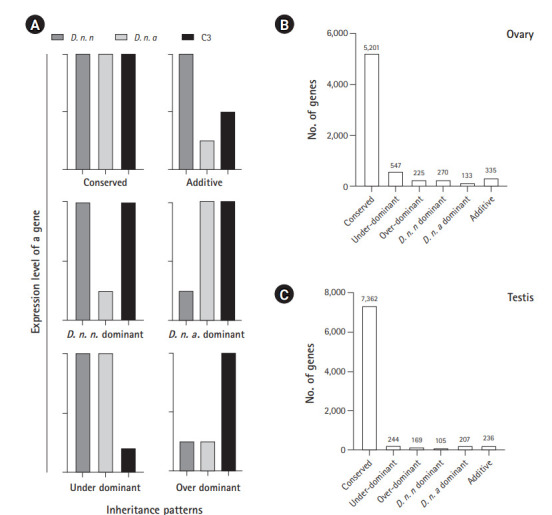
Inheritance patterns of gene expression levels deduced in C3 transcriptomes. (A) Representation of six inheritance patterns of gene expression levels. Conserved: gene which is not differentially expressed in C3 in comparison with both the parents and also not a DEG between the parental transcriptomes, Over-dominant: gene for which expression is significantly higher in the C3 than both parents, Under-dominant: gene for which expression is significantly lower in the C3 than both parent, Additive: gene which is differentially expressed in parents and show intermediate expression in C3, and *nasuta/albomicans* dominant: gene which is differentially expressed in C3 and is exhibiting similar patterns of expression with a corresponding parental lineage. *D. n. n* corresponds to *Drosophila nasuta nasuta* and *D. n. a* corresponds to *Drosophila nasuta albomicans*). (B) Expression inheritance deduced in the C3 ovary. (C) Expression inheritance deduced in the C3 testis. C3, Cytorace-3.

Among the 7,114 genes expressed in ovary and 8,673 genes expressed in testis of C3, conserved inheritance pattern was predominant for both testis (85%) and ovary (73%). A total of 3% of genes were under the additive category in the testis and 5% in the ovary, while the remaining genes were misexpressed in C3. Four hundred thirteen of the misexpressed genes in the testis and 772 in the ovary were either over- or under-dominant in C3. Number of genes following the *D. n. nasuta* dominance was twice (270 genes) when compared to *D. n. albomicans* dominance (133 genes) in the ovary. This trend was the opposite for testis transcriptome, where only 105 genes showed *D. n. nasuta* dominance and 207 showed *D. n. albomicans* dominance. Few genes that are over-/under-dominant in C3 also exhibit *D. n. nasuta*/ *D. n. albomicans* dominance. The results are summarized in Fig. 5.

### Validation of DEGs through RT-qPCR

**Fig. 6.**
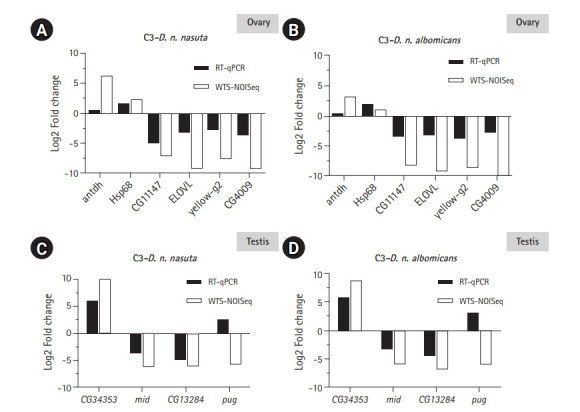
RT-qPCR validation of the transcriptome data. Log2-fold changes (Log2FC) between C3 and *Drosophila nasuta nasuta* ovaries (A), C3 and *Drosophila nasuta albomicans* ovaries (B), C3 and *D. n. nasuta* testis (C), and C3 and *D. n. albomicans* testis (D) are shown along Y axis for genes represented on X axis. White bars indicate Log2FC evaluated by Illumina sequencing data (WTS-NOISeq) and black bars indicate Log2FC derived from RT-qPCR. RT-qPCR results were in good agreement with Illumina sequencing analysis except for the gene *antdh* in the ovary and *pug* in testis which failed to match the WTS pattern of fold change. RT-qPCR, real-time quantitative polymerase chain reaction; C3, Cytorace-3; WTS, whole transcriptome sequencing.

Six DEGs each for ovary and testis were validated using RT-qPCR to check the reliability of the differential expression results quantified by WTS. Of the six, five genes closely tallied DEG analysis results conducted on ovarian Illumina sequencing data (Fig. 6A and 6B). One gene *antdh* was not differentially expressed in C3 relative to both the parents. Among the testis transcriptomes, three genes closely matched the DEG analysis results conducted on Illumina sequencing data (Fig. 6C and 6D). For the genes *E(spl)m3-HLH *and *CG3339* expression were detected only in the C3 sample. This trend was similar to WTS data recorded for these genes. Only gene *pug* did not coincide with the WTS data and was found upregulated in C3 than the parental testis transcriptomes. This demonstrated the reliability of the WTS technique to quantify gene expression. Overall, RT-qPCR validations were in good agreement with WTS data.

## Discussion

This study explores gene expression divergence patterns in an evolving population of a hybrid lineage, C3. This stable population of ~850 generations represents nearly 30 years of laboratory evolution. The results of our comparative transcriptome analyses with parental lineages revealed differential expression of genes in the gonadal tissues of C3. Besides, the expression divergence recorded in this model surpasses the expression divergence recorded between the parents in the ovarian (8%) [37] and testis transcriptomes (4%) (Supplementary Fig. 5). The genome of C3 being an admixture of the two parental genotypes emanated from chromosome recombination emphasizes the potential impact of hybridization on the genome, invariably leading to C3 acquiring unique and more divergent expression patterns in a brief duration.

Our analysis showed six inheritance patterns a gene could follow as a consequence of hybridization in a genome. A significant proportion of the conserved inheritance pattern of expressed genes observed in ovaries (73%) and testes (85%) of C3 is similar to earlier reports made in the fertile hybrids obtained with *D. pseudoobscura* species [13] and *D. mojavensis/ D. arizonae* [38] with a recent divergence history. With an estimated divergence time of 0.3-0.5 MY between *D. n. nasuta* and *D. n. albomicans*, the parental lineages of C3 equally represent a recently diverged sibling species pair with *D. n. albomicans* diverging from *D. n. nasuta* or nasuta like ancestor [39,40]. Therefore, the recorded proportion of conservation in gene expression could be attributed to the recent divergence of the parental races, further suggesting the process of hybridization has not affected these genes. Co-adaptation between cis and trans-regulatory networks is documented in sea urchins to mammals [41,42].

This co-adaptation might be maintaining the optimal gene expression levels in the conserved category. An asymmetrical pattern of expression divergence was observed in the non-conserved category. More genes in C3 were differentially expressed relative to the maternal parent *D. n. albomicans* in ovarian and testis transcriptomes. Among the non-conserved categories, a proportion followed *D. n. nasuta* dominance while a minor portion indicated *D. n. albomicans* dominance in the ovarian transcriptome, indicating that the alleles of these expressed genes might be from *D. n. nasuta* and is favored over *D. n. albomicans* in the ovaries and the testis, the trend was opposite with more genes showing *D. n. albomicans* dominance. A plausible explanation for this bidirectional preference could be due to the unequal amounts of parental genetic content found in C3. When C3 was initially derived, it contained precisely 50% genome from each parent, and in subsequent generations, parental chromosome selection coupled with recombination may have led to unequal contributions. This is evident in the present stabilized karyotype of C3, which resembles *D. n. nasuta* and comprising 40% of *D. n. albomicans* chromosomes. Differential expression can potentially influence phenotypic and trait changes [43], with the upregulation, mainly conferring benefits to an organism while the down-regulation precluding benefits. Assessment of functionality and pathway categorization of the common DEGs in ovaries and testis of C3 revealed a broader distribution of the genes across multiple pathways, majorly comprising metabolism-related processes. The number of genes distributed across metabolism-related pathways is primarily downregulated. The results hint that these genes might influence the short life span recorded for C3 [44]. Although this is speculative, the role of known candidate genes related to metabolism and aging needs a detailed investigation. Additionally, it provides an opportunity of identifying potential novel longevity-related genes in this model.

Based on existing research in C3, we have an understanding of divergence in this model. C3 differed from parental lineages and other cytoraces for key life-history related traits like lifetime female fecundity, lifetime fertility, and ovariole numbers, which represent indicative measures of population fitness [45]. These population differences could be attributed to the observed expression patterns as these traits are polygenic and mostly influenced by genes having a function in the ovary and testis. In *Drosophila*, nearly 76% of all the genes are expressed in the testis, and 47% of the genes are expressed in the ovaries providing the transcriptional diversity required to control the mechanisms of spermatogenesis and oogenesis, respectively [3]. With the amount of gene expression conservation that is quantified, it can be argued that the conserved genes are contributing to the normal functioning of the ovaries and testis in the C3. The quantified expression divergence could be contributing to the observed phenotypic variations, with the gonads seemingly tolerating the expression divergence.

Novel expression profiles can evolve in the hybrids when two independently evolving lineages are crossed together. The consequences of this co-evolution of the expression networks in the genetic background of a hybrid can be deleterious. Studies have shown various abnormalities affecting the reproductive system leading to complete sterility [46,47]. Regulatory incompatibility models have shown hybridization-induced profound effects at a transcription level with consequences like misexpression of genes in the F1 hybrids of *D. melanogaster* and *D. sechellia* [14]. Under-expression of genes with gonadal atrophy was recorded in female hybrids of D. melanogaster and D. simulans accompanied with significant over-expression of male-biased genes [15]. Significant misexpression for genes of spermatogenesis and reproductive proteases was seen in sterile hybrids of *Drosophila* [13,16]. Misexpression of regulatory factors and metabolic regulatory genes was observed in the hybrids of *Drosophila* [19]. However, alternatively, the co-evolution of expression networks in the hybrids might constitute novel and heritable gene expression patterns imparting beneficial effects. Evidence of altered gene expression levels in hybrids contributing to transgressive hybrid phenotypes is well documented in plants [48,49]. Over time, independently evolving lineages accumulate genetic variations in regulatory and coding sequences caused by genetic and environmental factors like a response mechanism to the evolutionary challenges imparted on an adapting organism. Even in hybrid populations, in which two parental genomes clash, such response mechanisms are expected to occur. Therefore, the accumulated genetic variations in a persisting hybrid population could have stemmed from such response mechanisms. Before evolving into an independent genetic entity, the hybrid products of *D. n. nasuta* and *D. n. albomicans* exhibited F1 heterosis and F2 breakdown for a few critical parameters of fitness [50]. Fertility tests in F2 and backcross progeny recorded a more considerable number of sterile males than females [50]. Fertile surviving recombinants reproduced, and with the gradual diminishment of karyotypic mosaicism, the stabilized forms became established populations [6,7].

Cytoraces are thought to evolve at a more accelerated rate due to hybridization and inbreeding, which may lead to faster fixation of variations than the natural populations. This accelerated accumulation of differences have been demonstrated through several molecular studies in cytoraces for increased genetic variability in inter-simple sequence repeat [51], nucleotide variations observed in *Sod1* and *Rpd3* genes, higher levels of RPD3 and SIR2 proteins [44], *always early (aly)* a meiotic arrest gene has shown eight times greater rate of substitutions amongst cytoraces than their parents and amongst species of subgenera [9]. Empirical evidence of assortative mating in the C3 males [52] is the first report from the *nasuta-albomicans* complex of *Drosophila*, making it a potential model for studying hybridization-induced behavioral trait evolution. The assortative mating contributes to premating isolation and is more likely to evolve if the parental species are intermediately diverged [20]. Though complete reproductive isolation from their parents has been unachieved in the cytoraces, especially in C3, they constitute a distinct lineage due to the mixed ancestry. Examples of such distinct hybrid taxa with incomplete reproductive barriers are reported in Oxford ragwort [53], Appalachian swallowtail butterflies [54], Cottus fishes [55], and Italian sparrows [56]. The available literature and the quantified expression divergence in C3 reflect the extent to which hybridization has altered the genetic architecture permitting a sustainable population.

In our attempt to profile the gonadal gene expressions in the laboratory evolved *Drosophila* hybrid lineage through comparative transcriptome approaches, we have recorded an accumulated expression divergence which surpasses that between parental lineages illustrating the strong impact of hybridization driving gene expression changes in a brief duration. This transcriptome dataset represents the first in an independently evolving homoploid hybrid lineage exhibiting incipient speciation in *Drosophila*. This documentation in the gonads prompts an extension from the whole organism's perspective and specific somatic tissues in this model. Besides, an evolutionarily unfinished product like C3 and in general cytoraces in their current incipient stages provides more information about the process of ongoing hybridization-induced changes than a finished evolutionary product. Our study contributes primarily toward the enrichment of genomic resources in the context of ecological speciation and provides an unprecedented global view of the transcriptomes for fundamental support in future research.

## Figures and Tables

**Table 1.** Summary of transcriptome assembly statistics showing results of assembly construction, structural annotations, and read mapping for the tissue-specific de novo assemblies of C3 and tissue-specific co-assemblies

	C3 ovary	C3 testis	Pooled ovary	Pooled testis
Trinity transcripts	16,642	20,823	25,129	31,473
Transcripts after clustering	15,273	19,465	15,273	19,465
Predicted coding regions (ORFs)	10,987	13,028	13,239	16,230
Mapped reads (%)	96.92	95.93	NA	NA
Average contig length (bp)	1,420	1,085.76	1,607.25	1,305.91
Total assembled bases	23,632,851	22,608,786	40,388,659	41,100,823
GC (%)	45.11	45.03	45.34	44.9
Contig N50 (bp)	2,399	1,740	2,593	2,030

C3, Cytorace-3; ORF, open reading frame; NA, not available.

**Table 2.** Summary of functional annotations generated for tissue-specific de novo assemblies of C3 using homology searches to various databases

Database	C3 ovary	C3 testis
Protein hits (Swiss-Prot BLASTP *Drosophilidae* only)	9,793	11,540
Protein hits (Swiss-Prot BLASTP)	8,133	10,247
Protein hits (Swiss-Prot BLASTX)	8,868	8,756
EggNOG database hits	198	3,734
Pfam	4,180	3,887
TmHMM predicted protein hits	1,711	1,983
SignalP hits	471	544

C3, Cytorace-3.

## Supplementary Materials

Supplementary Table 1. List of Forward and reverse primer sequences of genes used for RT-qPCR validations

Supplementary Table 2. List of significantly enriched KEGG pathways among the genes upregulated in C3 ovarian transcriptome against both parents

Supplementary Table 3. List of significantly enriched KEGG pathways among the genes downregulated in C3 ovarian transcriptome against both parents

Supplementary Table 4. List of significantly enriched KEGG pathways among the genes upregulated in C3 testis transcriptome against both parents

Supplementary Table 5. List of significantly enriched KEGG pathways among the genes downregulated in C3 testis transcriptome against both parents.

Supplementary Fig. 1. Gene ontology classification of commonly upregulated genes in C3 ovarian transcriptome. Representation of top 10 GO terms at level 2 classification for the three functional categories. GO categories are shown in the X axis and percentage of genes in the Y axis (Log scale). C3, Cytorace-3; GO, gene ontology.

Supplementary Fig. 2. Gene ontology classification of commonly downregulated genes in C3 ovarian transcriptome. Representation of top 10 Gene ontology terms at level 2 classification for the three functional categories. GO categories are shown in the X axis and percentage of genes in the Y axis (Log scale). C3, Cytorace-3; GO, gene ontology.

Supplementary Fig. 3. Gene ontology classification of commonly upregulated genes in C3 testis transcriptome. Representation of top 10 GO terms at level 2 classification for the three functional categories. GO categories are shown in the X axis and percentage of genes in the Y axis (Log scale). C3, Cytorace-3; GO, gene ontology.

Supplementary Fig. 4. Gene ontology classification of commonly downregulated genes in C3 testis transcriptome. Representation of top 10 GO terms at level 2 classification for the three functional categories. GO categories are shown in the X axis and percentage of genes in the Y axis (Log scale). C3, Cytorace-3; GO, gene ontology.

Supplementary Fig. 5. Summary plot for the relative expression of genes between *D. n. nasuta* (*D. n. n*) and *D. n. albomicans (D. n. a)*. (A) Relative expression of genes in the ovarian transcriptomes between *D. n. n* and D. n. a. A Total of 11,282 genes were considered expressed, of which 392 were upregulated in *D. n. n* and 541 genes were downregulated in *D. n. n* in comparison to *D. n. a.* (B) Relative expression of genes in the testis transcriptomes between *D. n. n* and *D. n. a.* A Total of 11,437 genes were considered expressed, of which 203 were upregulated in *D. n. n* and 214 genes were downregulated in *D. n. n* in comparison to *D. n. a.* Red points indicate significant differentially expressed genes and black points represent non-differentially expressed genes.
