# Supplementary material for: De novo assembly, annotation and gene expression profiles of gonads of Cytorace-3, a hybrid lineage of Drosophila nasuta nasuta and D. n. albomicans
Source: Genomics Inform. 2021 Mar 9;19(1):e8. doi: 10.5808/gi.20051 (PMC8042302; doi:10.5808/gi.20051)
Supplement: Supplementary Table 1. — List of Forward and reverse primer sequences of genes used for RT-qPCR validations [file gi-20051-suppl1.pdf]

Supplementary Table 1. List of Forward and reverse primer sequences of genes used for RT-qPCR validations

Gene	Forward primer 5′-3′	Forward primer Tm (°C)	Reverse primer 5′-3′	Reverse primer Tm (°C)
Gapdh2 (FBgn0001092)	GAAGGGCATTTTGGGCTACA	55.38	CGAACACCGAGGAGTGAGTGT	59.26
antdh (FBgn0026268)	ATGGGCATTGTGATGTGC	58.81	CGAGAATGCTGTTGATGAGC	59.55
Hsp68 (FBgn0001230)	TGAGTGCCAAGGAGATGG	58.19	ATGTCGGCTTGCGATAGG	59.78
CG11147 FBgn0031734)	AGTCCCCGATACCATTCG	58.30	TCATCAGCGCCTTTATGC	58.46
ELOVL (FBgn0037534)	TCTTCACATCGCCATTCG	59.28	CTTCTACAAGCGTGCCTACG	58.75
Yellow-g2 (FBgn0035328)	TCGCTGAAGTCCGAGTACC	58.97	TGCCAATGATGACCATGC	60.01
CG4009 (FBgn0038469)	TCATTGCCAAGCAGTTCC	51.93	TTGGGAGCGTGAGAAACC	53.6
CG34353 (FBgn0085382)	GCACTCATCCCAAATGTACG	59.00	GCATCAGATTGCCATAGCC	59.20
Mid (FBgn0261963)	CAGGATAACAAGCGCAAGG	59.43	TGGGATCCGCATTATTGG	60.24
CG13284 (*FBgn0032614)*	TGGCAAGGAATATGCAAGG	59.63	ACGGCAATCAGCTTCTCC	58.91
Pug (FBgn0020385)	ATGCGGACATCTTGATCG	58.06	GATGCCACAATCGATGACC	59.87
E(spl)m3-HLH (FBgn0002609)	GGCAAGGACGAACTAATCG	58.31	CGACTCTTGCGAACTGACC	59.56
CG3339 FBgn0039510)	GACGAACTCTTTGGCATCG	59.38	GGCATATTCGCCTGTTCC	59.03

RT-qPCR, real-time quantitative polymerase chain reaction
