# Supplementary material for: De novo assembly, annotation and gene expression profiles of gonads of Cytorace-3, a hybrid lineage of Drosophila nasuta nasuta and D. n. albomicans
Source: Genomics Inform. 2021 Mar 9;19(1):e8. doi: 10.5808/gi.20051 (PMC8042302; doi:10.5808/gi.20051)
Supplement: Supplementary Table 2. — List of significantly enriched KEGG pathways among the genes upregulated in C3 ovarian transcriptome against both parents [file gi-20051-suppl2.pdf]

Supplementary Table 2. List of significantly enriched KEGG pathways among the genes upregulated in C3 ovarian transcriptome against both parents

Sl. No.	KEGG pathway	Input number	Background number	p-value
1	Sulfur relay system	3	8	0.000233639
2	Insect hormone biosynthesis	3	17	0.001495383
3	Phototransduction - fly	3	34	0.008830346
4	Drug metabolism - cytochrome P450	3	56	0.030750138
5	Metabolism of xenobiotics by cytochrome P450	3	57	0.032101471
6	Longevity regulating pathway - multiple species	3	57	0.032101471
7	Hippo signaling pathway - fly	3	59	0.034896026
8	Apoptosis - fly	3	66	0.045628791
9	ABC transporters	2	20	0.02635292
10	Apoptosis - multiple species	2	23	0.033463719

Databases: KEGG PATHWAY, Statistical test method: hypergeometric test/Fisher exact test, FDR correction method: Benjamini and Hochberg.

KERGG, Kyoto Encyclopedia of Genes and Genomes; C3, Cytorace-3.
