# Supplementary material for: De novo assembly, annotation and gene expression profiles of gonads of Cytorace-3, a hybrid lineage of Drosophila nasuta nasuta and D. n. albomicans
Source: Genomics Inform. 2021 Mar 9;19(1):e8. doi: 10.5808/gi.20051 (PMC8042302; doi:10.5808/gi.20051)
Supplement: Supplementary Table 3. — List of significantly enriched KEGG pathways among the genes downregulated in C3 ovarian transcriptome against both parents [file gi-20051-suppl3.pdf]

**Supplementary Table 3.** List of significantly enriched KEGG pathways among the genes downregulated in C3 ovarian transcriptome against both parents

| Sl. No. | KEGG pathway | Input number | Background number | p-value |
|---|---|---|---|---|
| 1 | Hippo signaling pathway - fly | 12 | 59 | 1.30E-05 |
| 2 | MAPK signaling pathway - fly | 11 | 94 | 0.002093644 |
| 3 | FoxO signaling pathway | 7 | 62 | 0.015371518 |
| 4 | Lysine degradation | 6 | 37 | 0.005343607 |
| 5 | RNA degradation | 6 | 58 | 0.03389575 |
| 6 | TGF-beta signaling pathway | 5 | 41 | 0.029351392 |
| 7 | Hippo signaling pathway - multiple species | 4 | 16 | 0.006101408 |

Databases: KEGG PATHWAY, Statistical test method: hypergeometric test/Fisher exact test, FDR correction method: Benjamini and Hochberg.

KEGG, Kyoto Encyclopedia of Genes and Genomes; C3, Cytorace-3; MAPK, mitogen-activated protein kinase; TGF, transforming growth factor.
