# Supplementary material for: De novo assembly, annotation and gene expression profiles of gonads of Cytorace-3, a hybrid lineage of Drosophila nasuta nasuta and D. n. albomicans
Source: Genomics Inform. 2021 Mar 9;19(1):e8. doi: 10.5808/gi.20051 (PMC8042302; doi:10.5808/gi.20051)
Supplement: Supplementary Table 4. — List of significantly enriched KEGG pathways among the genes upregulated in C3 testis transcriptome against both parents [file gi-20051-suppl4.pdf]

Supplementary Table 4. List of significantly enriched KEGG pathways among the genes upregulated in C3 testis transcriptome against both parents

Sl. No.	KEGG pathway	Input number	Background number	p-value
1	ECM-receptor interaction	3	82	0.001124796
2	2-Oxocarboxylic acid metabolism	2	18	0.001038718
3	Glutathione metabolism	2	52	0.007422237
4	Platinum drug resistance	2	75	0.01464848
5	Biosynthesis of amino acids	2	75	0.01464848
6	Protein digestion and absorption	2	90	0.020479721
7	Pyrimidine metabolism	2	105	0.027115577
8	Lysosome	2	123	0.036052812
9	Tight junction	2	139	0.044811712
10	Glycosaminoglycan biosynthesis - keratan sulfate	1	15	0.037470894

Databases: KEGG PATHWAY, Statistical test method: hypergeometric test/Fisher exact test, FDR correction method: Benjamini and Hochberg.

KEGG, Kyoto Encyclopedia of Genes and Genomes; C3, Cytorace-3; ECM, extracellular matrix.
