# Supplementary material for: De novo assembly, annotation and gene expression profiles of gonads of Cytorace-3, a hybrid lineage of Drosophila nasuta nasuta and D. n. albomicans
Source: Genomics Inform. 2021 Mar 9;19(1):e8. doi: 10.5808/gi.20051 (PMC8042302; doi:10.5808/gi.20051)
Supplement: Supplementary Table 5. — List of significantly enriched KEGG pathways among the genes downregulated in C3 testis transcriptome against both parents. [file gi-20051-suppl5.pdf]

Supplementary Table 5. List of significantly enriched KEGG pathways among the genes downregulated in C3 testis transcriptome against both parents.

Sl. No.	KEGG pathway	Input number	Background number	p-value
1	Metabolic pathways	33	1243	2.62E-18
2	Drug metabolism - other enzymes	7	46	1.41E-09
3	Pyrimidine metabolism	7	105	2.74E-07
4	Circadian entrainment	5	95	4.35E-05
5	Proximal tubule bicarbonate reclamation	4	23	3.40E-06
6	Dorso-ventral axis formation	4	28	6.87E-06
7	Pyruvate metabolism	4	40	2.50E-05
8	Glutathione metabolism	4	52	6.53E-05
9	Long-term depression	4	60	0.000110291
10	Amphetamine addiction	4	67	0.000165189
11	Bile secretion	4	71	0.0002042
12	Retrograde endocannabinoid signaling	4	101	0.000734776
13	Glutamatergic synapse	4	114	0.001135083
14	Dopaminergic synapse	4	130	0.001811747
15	cAMP signaling pathway	4	199	0.007913681
16	MicroRNAs in cancer	4	299	0.02966777
17	PI3K-Akt signaling pathway	4	342	0.044686194

18	Nicotine addiction	3	40	0.000609084
19	Fat digestion and absorption	3	41	0.000651821
20	Cocaine addiction	3	49	0.001063577
21	N-Glycan biosynthesis	3	49	0.001063577
22	Arginine and proline metabolism	3	50	0.001124241
23	Amyotrophic lateral sclerosis (ALS)	3	51	0.001187046
24	Long-term potentiation	3	66	0.002404487
25	Glycolysis / Gluconeogenesis	3	67	0.00250515
26	Drug metabolism - cytochrome P450	3	69	0.00271421
27	PPAR signaling pathway	3	72	0.003047454
28	Metabolism of xenobiotics by cytochrome P450	3	73	0.00316386
29	Chemical carcinogenesis	3	82	0.004335122
30	Pancreatic secretion	3	96	0.006621559
31	Glucagon signaling pathway	3	102	0.007783206
32	Purine metabolism	3	176	0.031832183
33	Other glycan degradation	2	18	0.00265081
34	Renin-angiotensin system	2	23	0.004133282
35	Vitamin digestion and absorption	2	24	0.004466506
36	Glycosaminoglycan biosynthesis - heparan sulfate / heparin	2	24	0.004466506

37	Citrate cycle (TCA cycle)	2	30	0.006714873
38	Galactose metabolism	2	31	0.007130224
39	Pentose and glucuronate interconversions	2	36	0.009375512
40	Tryptophan metabolism	2	40	0.011368553
41	Sphingolipid metabolism	2	47	0.015259185
42	Amino sugar and nucleotide sugar metabolism	2	48	0.015855461
43	Ovarian steroidogenesis	2	50	0.017077522
44	Mineral absorption	2	52	0.018338376
45	Starch and sucrose metabolism	2	57	0.021655931
46	Steroid hormone biosynthesis	2	58	0.022347156
47	Glycerolipid metabolism	2	59	0.023047433
48	Retinol metabolism	2	65	0.027434824
49	Adipocytokine signaling pathway	2	70	0.031325782
50	Platinum drug resistance	2	75	0.035420243
51	Aldosterone synthesis and secretion	2	81	0.040589509
52	Hematopoietic cell lineage	2	88	0.046954118
53	Ribosome biogenesis in eukaryotes	2	89	0.047891427
54	Protein digestion and absorption	2	90	0.048835557
55	Sulfur metabolism	1	10	0.041372824
56	Ubiquinone and other terpenoid-quinone	1	11	0.045048579

	biosynthesis

Databases: KEGG PATHWAY, Statistical test method: hypergeometric test / Fisher's exact test, FDR correction method: Benjamini and Hochberg.

KEGG, Kyoto Encyclopedia of Genes and Genomes; PI3K, phosphoinositide 3-kinase.
