# Supplementary material for: De novo assembly, annotation and gene expression profiles of gonads of Cytorace-3, a hybrid lineage of Drosophila nasuta nasuta and D. n. albomicans
Source: Genomics Inform. 2021 Mar 9;19(1):e8. doi: 10.5808/gi.20051 (PMC8042302; doi:10.5808/gi.20051)
Supplement: Supplementary Fig. 3. — Gene ontology classification of commonly upregulated genes in C3 testis transcriptome. Representation of top 10 GO terms at level 2 classification for the three functional categories. GO categories are shown in the X axis and percentage of genes in the Y axis (Log scale). C3, Cytorace-3; GO, gene ontology. [file gi-20051-suppl8.pdf]

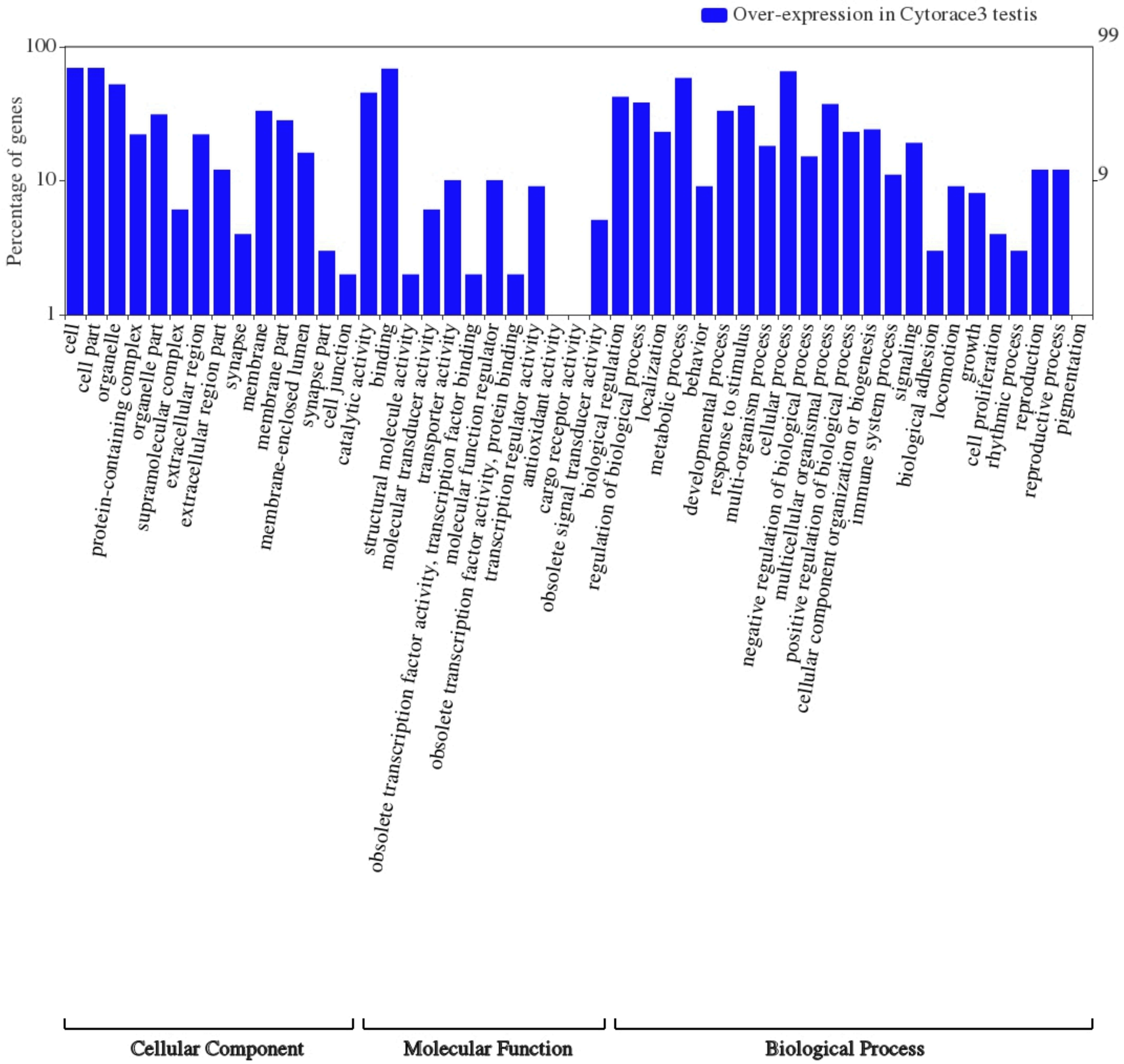

Supplementary Fig. 3. Gene ontology classification of commonly upregulated genes in C3 testis transcriptome. Representation of top 10 GO terms at level 2 classification for the three functional categories. GO categories are shown in the X axis and percentage of genes in the Y axis (Log scale). C3, Cytorace-3; GO, gene ontology.
