# Supplementary material for: De novo assembly, annotation and gene expression profiles of gonads of Cytorace-3, a hybrid lineage of Drosophila nasuta nasuta and D. n. albomicans
Source: Genomics Inform. 2021 Mar 9;19(1):e8. doi: 10.5808/gi.20051 (PMC8042302; doi:10.5808/gi.20051)
Supplement: Supplementary Fig. 5. — Summary plot for the relative expression of genes between D. n. nasuta (D. n. n) and D. n. albomicans (D. n. a). (A) Relative expression of genes in the ovarian transcriptomes between D. n. n and D. n. a. A Total of 11,282 genes were considered expressed, of which 392 were upregulated in D. n. n and 541 genes were downregulated in D. n. n in comparison to D. n. a. (B) Relative expression of genes in the testis transcriptomes between D. n. n and D. n. a. A Total of 11,437 genes were considered expressed, of which 203 were upregulated in D. n. n and 214 genes were downregulated in D. n. n in comparison to D. n. a. Red points indicate significant differentially expressed genes and black points represent non-differentially expressed genes. [file gi-20051-suppl10.pdf]

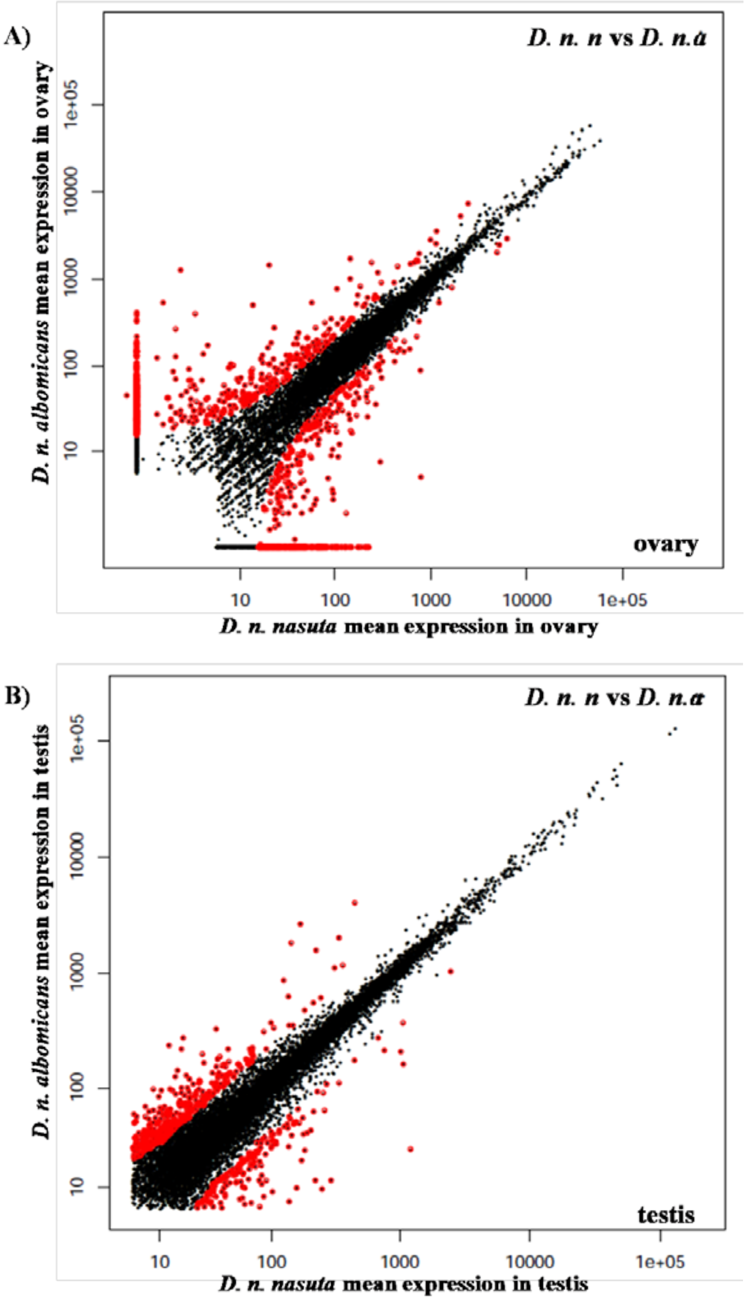

Supplementary Fig. 5. Summary plot for the relative expression of genes between *D. n. nasuta* (*D. n. n*) and *D. n. albomicans* (*D. n. a*). (A) Relative expression of genes in the ovarian transcriptomes between *D. n. n* and *D. n. a*. A Total of 11,282 genes were considered expressed, of which 392 were upregulated in *D. n. n* and 541 genes were downregulated in *D. n. n* in comparison to *D. n. a*. (B) Relative expression of genes in the testis transcriptomes between *D. n. n* and *D. n. a*. A Total of 11,437 genes were considered

expressed, of which 203 were upregulated in *D. n. n* and 214 genes were downregulated in *D. n. n* in comparison to *D. n. a*. Red points indicate significant differentially expressed genes and black points represent non-differentially expressed genes.
